# C_60_ Fullerene Prevents Restraint Stress-Induced Oxidative Disorders in Rat Tissues: Possible Involvement of the Nrf2/ARE-Antioxidant Pathway

**DOI:** 10.1155/2018/2518676

**Published:** 2018-11-13

**Authors:** Olga O. Gonchar, Andriy V. Maznychenko, Nataliya V. Bulgakova, Inna V. Vereshchaka, Tomasz Tomiak, Uwe Ritter, Yuriy I. Prylutskyy, Iryna M. Mankovska, Alexander I. Kostyukov

**Affiliations:** ^1^Bogomoletz Institute of Physiology, Bogomoletz Str. 4, Kyiv 01024, Ukraine; ^2^Gdansk University of Physical Education and Sport, Kazimierza Gorskiego Str. 1, 80-336 Gdansk, Poland; ^3^Institute of Chemistry and Biotechnology, Technical University of Ilmenau, Weimarer Str. 25, Ilmenau 98693, Germany; ^4^Taras Shevchenko National University of Kyiv, ESC “Institute of Biology and Medicine”, Volodymyrska Str. 64, Kyiv 01601, Ukraine

## Abstract

The effects of C_60_FAS (50 and 500 *μ*g/kg) supplementation, in a normal physiological state and after restraint stress exposure, on prooxidant/antioxidant balance in rat tissues were explored and compared with the effects of the known exogenous antioxidant N-acetylcysteine. Oxidative stress biomarkers (ROS, O_2_·^−^, H_2_O_2_, and lipid peroxidation) and indices of antioxidant status (MnSOD, catalase, GPx, GST, *γ*-GCL, GR activities, and GSH level) were measured in the brain and the heart. In addition, protein expression of Nrf2 in the nuclear and cytosol fractions as well as the protein level of antiradical enzyme MnSOD and GSH-related enzymes *γ*-GCLC, GPx, and GSTP as downstream targets of Nrf2 was evaluated by western blot analysis. Under a stress condition, C_60_FAS attenuates ROS generation and O_2_·^−^ and H_2_O_2_ releases and thus decreases lipid peroxidation as well as increases rat tissue antioxidant capacity. We have shown that C_60_FAS supplementation has dose-dependent and tissue-specific effects. C_60_FAS strengthened the antiradical defense through the upregulation of MnSOD in brain cells and maintained MnSOD protein content at the control level in the myocardium. Moreover, C_60_FAS enhanced the GSH level and the activity/protein expression of GSH-related enzymes. Correlation of these changes with Nrf2 protein content suggests that under stress exposure, along with other mechanisms, the Nrf2/ARE-antioxidant pathway may be involved in regulation of glutathione homeostasis. In our study, in an *in vivo* model, when C_60_FAS (50 and 500 *μ*g/kg) was applied alone, no significant changes in Nrf2 protein expression as well as in activity/protein levels of MnSOD and GSH-related enzymes in both tissues types were observed. All these facts allow us to assume that in the *in vivo* model, C_60_FAS affects on the brain and heart endogenous antioxidative statuses only during the oxidative stress condition.

## 1. Introduction

Today, everybody of the living organism is exposed to stress of various origins. Numerous researches indicate that the most harmful effects to an organism result from social and psychological factors [1, 2]. Emotional overstrain as one of the types of stress states is constantly encountered not only in daily life but also as an imperative component of such human activities as sports, military, and aerospace. Acute stress of sufficient strength threatens body homeostasis, which results in biochemical and physiological disturbances leading to serious health risks [2–4]. Exposure to a stressful situation is well known to stimulate various damaging pathways, causing overproduction of free radicals such as superoxide anion (O_2_·^−^), hydroxyl radical (OH·), and nonradical hydrogen peroxide (H_2_O_2_), leading to lipid peroxidation, protein oxidation, DNA damage, and cell death [5]. In response to oxidative attack, cells have developed an antioxidant defense system to maintain cellular redox homeostasis and to protect cells from injury [6, 7]. Classical antioxidant enzymes including superoxide dismutase (SOD), catalase, and glutathione peroxidase (GPx) as well as small thiol-containing compound glutathione (GSH) may directly inactivate ROS and prevent ROS-initiated reactions [8]. Intracellular glutathione is the most abundant nonprotein thiol with several vital functions such as direct scavenging of free radicals, detoxification of electrophilic compounds, and modulation of cellular redox status and thiol-disulphide status of proteins, as well as regulation of cell signaling and repair pathways [9]. GSH homeostasis is modulated by self-adjusting the balance among GSH synthesis, utilization, and recycling, and the disturbances of these processes may cause an oxidant/antioxidant imbalance. Antioxidants of indirect action include so-called phase II detoxifying enzymes, which contribute to biosynthesis/recycling of thiols and facilitate excretion of oxidized, reactive secondary metabolites (e.g., quinones, epoxides, aldehydes, and peroxides) through reduction/conjugation reactions. They are represented by glutathione-S-transferase (GST) isozymes, NADPH: quinone oxidoreductase (NQO1), heavy (catalytic) and light (modifier) subunits of *γ*-glutamate-cysteine ligase (*γ*-GCL), glutathione peroxidase (GPx), and glutathione reductase (GR) and stress response proteins such as heme oxygenase- (HO-) 1 and heavy (FTH) and light (FTL) chains of ferritin [10].

Restraint is the one specific and classic method to simultaneously induce emotional and physiological stresses. Therefore, restraint is widely used for modulation of oxidative stress-induced pathological states [11]. Studies from several laboratories have demonstrated that acute restraint stress significantly elevated oxidative status and lipid peroxidation through downregulation of antioxidative enzymes, with glutathione depletion that disrupted the cellular redox state [12–15]. Restraint stress can affect central nervous system functions by producing neurochemical and hormonal disorders associated with imbalance in a prooxidant/antioxidant system [16, 17].

Numerous reports have shown that negative consequences of stress are amenable to improvement by exogenous antioxidant acting through various pathways to enhance resistance to stress exposure [10, 12, 18]. Indeed, pharmacological interventions targeting cellular endogenous antioxidants may be a promising stress management strategy for protecting against oxidative stress-induced cell damage. Fullerene (C_60_) is known as a spherical carbon molecule with a unique cage structure and is characterized as a strong radical sponge [19]. The antioxidant level of C_60_ has been reported to be several hundredfold higher than the level of other antioxidants [20, 21]. A great deal of information has accumulated concerning the beneficial effects of fullerene and its water-soluble derivatives, which include neuroprotection, radioprotection, antiproliferative, antitumor, and anti-inflammatory activities [22–25], but little is known of C_60_ protective effects and mechanisms of action in rat tissues under restraint stress condition.

Fullerene pretreatment has been known to prevent oxidative stress deleterious effects by direct ROS scavenging and/or increasing the antioxidant enzyme activities [20, 26, 27]. However, the mechanism involved in the cell protective actions of C_60_ is still open for discussion. Recently, polyhydroxylated derivatives of fullerene were demonstrated to induce endogenous phase II antioxidant enzymes via Nrf2/ARE-dependent mechanisms and attenuate oxidative stress-mediated cell death, thus providing new insights into the mechanisms of the antioxidant properties of fullerene [28, 29]. NF-E2-related factor 2 (Nrf2) is a member of the cap‘n'collar family of bZIP transcription factors that bind to the antioxidant response element (ARE) and thereby regulate the induction of genes encoding antioxidant proteins and phase II detoxifying enzymes. Nrf2 activation in response to oxidative injury has been shown to protect cells and tissues from oxidative stress [30]. Under normal conditions, Nrf2 is localized in the cytoplasm where it binds with the Keap1, which functions as an adaptor for Cul3-based E3 ligase to regulate the proteasomal degradation of Nrf2. However, after direct attack by ROS or resulting indirect actions such as phosphorylation, Nrf2 dissociates from Keap1 and thereby translocates into the nucleus and transactivates its target genes through ARE [31]. The coordinated transcriptional activation of the Nrf2-mediated antioxidant and prosurvival enzymes is a potential mechanism to maintain redox homeostasis and to abolish the deleterious effects of oxidative stress [32].

Therefore, our aim was to examine the effects of C_60_FAS in comparison with the actions of the known exogenous antioxidant N-acetylcysteine [33] on prooxidant/antioxidant homeostasis in rats in a normal physiological state and following restraint stress exposure. Many studies have shown that restraint stress exerts a profound impact on organs with a high metabolic level and the highest mass-specific oxygen consumption rates in the body such as the brain and the heart. Since the association of restraint stress with several neurodegenerative and cardiovascular diseases is well-documented [1–3], we investigated restraint stress-induced oxidative damage in the brain as well as in the heart as an important stress-sensitive peripheral organ.

Nrf2 is known to control both the basal and inducible expressions of genes encoding the heavy and light chains of *γ*-GCL, one of the important enzymes in GSH biosynthesis, and thereby takes part in the regulation of GSH recycling [10, 34]. However, whether and how Nrf2 modulates GSH homeostasis in rat tissues after C_60_FAS supplementation and following restraint stress exposure are poorly understood.

To further investigate the potential mechanisms of C_60_FAS-mediated protection against oxidative stress induced by restraint exposure, we examined protein expression of Nrf2 in the nuclear and cytosol fractions. In addition, the protein level of the antiradical enzyme MnSOD and GSH-related enzymes as downstream targets of Nrf2 was evaluated by western blot analysis.

## 2. Materials and Methods

### 2.1. Material Preparation and Characterization

A highly stable pristine C_60_ fullerene aqueous colloid solution (C_60_FAS) with purity of more than 99.96% has been prepared and characterized [27, 35]. This method is based on transferring fullerene from organic solution into the aqueous phase by ultrasonic treatment. The purity of the prepared C_60_FAS (i.e., the presence/absence of any residual impurities such as carbon black, toluene phase) was determined by HPLC analysis. The state of C_60_ fullerene in aqueous solution was monitored using atomic force microscopy. Small-angle neutron scattering measurements were carried out at the YuMO small-angle diffractometer at the IBR-2 pulsed reactor in the time-of-flight mode with a two-detector setup. The maximal concentration of C_60_ fullerenes in water obtained by this method was 0.15 mg/ml. Concentrated C_60_FAS contains both single C_60_ and its labile nanoassociates with sizes of 3–70 nm.

### 2.2. Experimental Design and Sample Collection

Male Wistar rats weighing 220–260 g were used in the study. The rats were housed in Plexiglas cages (4 rats per cage) and maintained in an air-filtered and temperature-controlled (20–22°C) room. Rats received a standard pellet diet and water ad libitum. The use of the animals was approved by the Ethics Committee of the Institute, and the study was performed in accordance with the European Communities Council Directive of 24 November 1986 (86/609/EEC). All chemicals were purchased from Sigma, Fluka, and Merck and were of the highest purity.

The study was performed in two phases. Phase I aimed at investigating the effect of C_60_FAS at various doses and N-acetylcysteine (NAC) as a positive control on markers of prooxidant/antioxidant balance in the whole brain and heart tissues. Rats were divided into four groups (*n* = 8/group) based on treatment: control/vehicle (C), fullerene-treated at a dose of 50 *μ*g/kg (F1), fullerene-treated at a dose of 500 *μ*g/kg (F2), and NAC-treated at a dose of 100 mg/kg in physiological saline (NAC). Animals were treated with F1, F2, or NAC intraperitoneally (i.p.) once daily for 1 week. The controls/vehicles were administrated i.p. with an equal volume of physiological saline during the same time period. The NAC was used according to previous study [36] that described the effective dose in similar experiments with animals. The C_60_FAS dosage was based on previous studies where the safety profile was checked and found to be nonlethal. No toxic effects or death has been fixed under the action of C_60_FAS after their oral administration to rats in the total dosage of 2 mg/kg for 5 days [21] and i.p. injection in the dose of 0.15 mg/kg [27].

Phase II was an acute restraint stress model alone and after pretreatment with the above-mentioned drugs. The experimental groups were as follows: group 1 received vehicle (physiological saline) and served as a control (1); group 2 was exposed to restraint for 6 h, representing an acute stressor (AS); group 3 was exposed to AS and received C_60_FAS (50 *μ*g/kg) (AS + F1); group 4 was subjected to AS and received C_60_FAS (500 *μ*g/kg) (AS + F2); group 5 was subjected to AS and received NAC (100 mg/kg) (AS + NAC). Drugs were administered i.p. between 9:00 a.m. and 10:00 a.m. once a day for 1 week prior to the restraint exposure. To habituate the rats to i.p. injection, all rats in groups 1 and 2 were administered physiological saline (1 ml/kg) daily for 1 week before the impact. The restraint was performed using a plastic rodent restrainer that allowed for a close fit to the rats. Experiments with the acute stressor were performed between 8:00 and 10:00 a.m. to minimize possible hormonal interference by circadian rhythms. During restraint stress, animals were not physically compressed, but food and water were deprived. Animals were decapitated immediately after the restraint sessions. At the time of sacrifice, the animals were lightly anesthetized with ether.

The brain and heart were rapidly removed, washed in ice-cold sterile physiological saline (0.9%), and a 10% homogenate (50 mM sodium phosphate buffer with 2 mM EDTA, pH 7.4) was prepared. Cellular debris was removed by centrifugation at 15,000g (4°C, 15 min), and supernatants were stored at −70°C. The protein concentration was estimated using the Bradford method with bovine serum albumin as a standard.

### 2.3. Evaluation of Oxidative Stress Markers

#### 2.3.1. Corticosterone Analysis

Whole blood, removed under ether anesthesia, was collected into heparin-coated test tubes, centrifuged at 1500g for 10 min at 4°C to separate plasma from erythrocytes and kept at –70°C until assayed. Plasma corticosterone was quantified using a commercial Demeditec Corticosterone rat/mouse ELISA kit (DEV9922, version 7-06/17, Demeditec Diagnostics, Germany) according to the manufacturer's instructions. The resulting concentration of plasma corticosterone was expressed as ng/ml using a corticosterone standard curve.

#### 2.3.2. Measurement of Reactive Oxygen Species (ROS) Formation

The data on ROS formation were obtained from dichlorofluorescein (DCF) fluorescence [37]. The tissue homogenates were loaded for 20 min at 37°C with a nonfluorescent probe (2′,7′-dichlorodihydrofluorescein diacetate, DCFH-DA) which is known to be decomposed in cells to give dichlorofluorescein upon oxidation by ROS, primarily hydroperoxide and superoxide anion. The final concentration of DCFH-DA was 5 mM. DCF formation was followed at the excitation wavelength of 488 nm and emission wavelength of 525 nm for 30 min by using a Hitachi F-2000 fluorescence spectrometer. The rate of DCFH-DA conversion to DCF was linear for at least 60 min, corrected with the autooxidation rate of DCFH-DA without protein. All assays were carried out in duplicate. Fluorescence was expressed as arbitrary fluorescence units.

#### 2.3.3. Measurement of Superoxide Radical Production

Tissue superoxide production was measured by superoxide dismutase-sensitive ferricytochrome *c* reduction assays, as described previously [38]. Briefly, equal portions of tissue homogenate (0.5 mg of protein) were incubated with 50 *μ*M acetylated ferricytochrome *c* in the presence or absence of superoxide dismutase (400 U/ml). To further ensure that any reduced ferricytochrome *c* is not reoxidized, catalase (125 U/ml) was added to the reaction, which removes any H_2_O_2_ formed. After incubation at 37°C for 15 min, reactions were terminated by adding 1 mM N-ethylmaleimide. Reduction of ferricytochrome *c* was measured by reading absorbance at 550 nm in a spectrophotometer. The amount of O_2_·^−^ release was calculated by dividing the difference in absorbance of the samples with and without superoxide dismutase by the extinction coefficient for the change from ferricytochrome *c* to ferrocytochrome *c* (*ε* = 24 mM^−1^ cm^-l^), and the results are expressed as nmol/min/mg protein.

#### 2.3.4. Measurement of Hydroperoxide

The H_2_O_2_ concentration in the tissue homogenates was measured using the FOX method, which is based on the peroxide-mediated oxidation of Fe^2+^, followed by the reaction of Fe^3+^ with xylenol orange (*o*-cresolsulphonephthalein 3′,3^″^-bis [methylimino] diacetic acid, sodium salt). This method is extremely sensitive and is used to measure low levels of water-soluble hydroperoxide present in the aqueous phase. To determine the H_2_O_2_ concentration, 500 *μ*l of the incubation medium was added to 500 *μ*l of the assay reagent (500 *μ*M ammonium ferrous sulphate, 50 mM H_2_SO_4_, 200 *μ*M xylenol orange, and 200 mM sorbitol). The absorbance of the Fe^3+^-xylenol orange complex (A560) was detected after 45 min. Standard curves of H_2_O_2_ were obtained for each independent experiment by adding variable amounts of H_2_O_2_ to 500 *μ*l of basal medium mixed with 500 *μ*l of the assay reagent. The data were normalized and are expressed as *μ*M H_2_O_2_ per mg of protein [39].

#### 2.3.5. Lipid Peroxidation Assay

Lipid peroxidation was measured from the formation of thiobarbituric acid-reactive substances (TBARS) using the method of Buege and Aust [40]. TBARS were isolated by boiling tissue homogenates for 15 min at 100°C with the thiobarbituric acid reagent (0.5% 2-thiobarbituric acid/10% trichloroacetic acid/0.63 mM hydrochloric acid) and measuring the absorbance at 532 nm. The results are expressed as nM/mg of protein using *ε* = 1.56 × 10^5^ mM^−1^ cm^−1^.

### 2.4. Evaluation of Biochemical Parameters

#### 2.4.1. Enzyme Activity Assay

Manganese superoxide dismutase (MnSOD) activity was measured by the method of Misra and Fridovich [41], based on the inhibition of autooxidation of adrenaline to adrenochrome by SOD contained in the examined samples. The samples were preincubated at 0°C for 60 min with 6 mM KCN, which produces total inhibition of Cu, Zn-SOD activity. The results are expressed as specific activity of the enzyme in units per mg protein. One unit of SOD activity was defined as the amount of protein causing 50% inhibition of the conversion rate of adrenaline to adrenochrome under specified conditions.

Catalase activity was measured by the decomposition of hydrogen peroxide, determined by a decrease in the absorbance at 240 nm [42].

*γ*-Glutamylcysteinyl ligase (*γ*-GCL) activity was determined following the method described by Seelig and Meister [43]. Briefly, enzyme activity was assayed at 37°C in reaction mixtures containing 0.1 M Tris-HCl buffer (pH 8.2), 0.15 M KCl, 5 mM ATP, 2 mM phosphoenolpyruvate, 10 mM glutamate, 10 mM *γ*-aminobutyrate, 20 mM MgCl_2_, 2 mM EDTA, 0.2 mM NADH, 17 mg pyruvate kinase, and 17 mg lactate dehydrogenase. The reaction was initiated by adding the sample, and the rate of decrease in absorbance at 340 nm was monitored. Enzyme-specific activity was measured as *μ*M of NADH oxidized per minute per milligram protein.

Activity of selenium-dependent glutathione peroxidase (GPx**)** was determined according to the method of Flohé and Gunzler [44]. Briefly, the reaction mixtures consisted of 50 mM KPO_4_ (pH 7.0), 1 mM EDTA, 1 mM NaN_3_, 0.2 mM NADPH, 1 mM GSH, 0.25 mM H_2_O_2_, and 226 U/ml glutathione reductase, and rates of NADPH oxidation followed at 340 nm.

The glutathione reductase (GR) reaction mixture contained phosphate buffer (0.2 M) pH 7.0, EDTA (2 mM), NADPH (2 mM), and GSSG (20 mM). The reaction is initiated by the addition of the sample, and the decrease in absorbance at 340 nm is followed at 30°C [45].

Glutathione-S-transferase (GST) activity was determined by assaying 1-chloro-2, 4-dinitrobenzene (CDNB) conjugation with GSH, as described by Warholm et al. [46]. The working solution contained 20 mM CDNB, 20 mM GSH, and 1 mM EDTA in 200 mM phosphate buffer. The conjugated product was recorded at 340 nm continuously for 5 min at 30°C (*ε* = 9.6 × 10^3^ M^−1^ cm^−1^).

#### 2.4.2. Measurement of the Reduced Glutathione Level

The reduced glutathione (GSH) was determined as described [47]. The tissue sample was mixed with sulphosalicylic acid (4%) and incubated at 4°C for 30 min. Thereafter, the mixture was centrifuged at 1200g for 15 min at 4°C, and 0.1 ml of this supernatant was added to phosphate buffer (0.1 M, pH 7.4) containing DTNB in abs. ethanol. The yellow color that developed was read immediately at 412 nm. The GSH content was calculated as nM GSH/mg of protein (*ε* = 13.6 × 10^3^ M^−1^ cm^−1^).

### 2.5. Western Blot Analysis

For immunoblotting analysis, cytosolic and nuclear proteins were extracted from the blood-free (heparin was injected, and buffer was perfused in situ) heart and brain as was described previously [48]. Briefly, the tissues were homogenized in ice-cold lysis buffer containing 10 mM HEPES (pH 7.9), 1.5 mM MgCl_2_, 10 mM KCl, 1 mM dithiothreitol, 0.1 mM EDTA, and 0.2 mM phenylmethylsulphonyl fluoride (PMSF) plus 1 *μ*g/ml Halt™ Protease and Phosphatase Inhibitor Cocktail (78440, Thermo Scientific Inc., USA). This suspension was incubated on ice for 15 min. Then, 12.5 *μ*l of 10% Nonidet P-40 was added, and the mixture was vigorously vortexed for 15 s. The cytoplasmic and nuclear fractions were separated by centrifugation at 15,000g at 4°C for 2 min. Equal amounts of protein were mixed with Laemmli buffer (S3401, Sigma), heated (99°C, 5 min), and then loaded onto 10–12% SDS polyacrylamide gels. Separated proteins were transferred onto polyvinylidene difluoride (PVDF) membranes, which were blocked in 5% nonfat milk in Tris-buffered saline-Tween (TBS-T) for 1 h at room temperature. Primary antibodies were applied overnight at 4°C. After washing in 1% nonfat milk in TBS-T, membranes were incubated with a secondary antibody conjugated to horseradish peroxidase (HRP) for 1 h at room temperature. Each antigen-antibody complex was visualized by the amino-ethylcarbazole reaction. The relative expression of the proteins was quantified using densitometric scanning and analyzed by ImageJ and is expressed as a percent of controls. All samples were analyzed at least twice, and the average value was calculated for each sample. *β*-Actin and Lamin B1 were used as loading controls. Antibodies and dilutions were as follow: Nrf2 1 : 1000 (Sigma-Aldrich Co.), MnSOD 1 : 500 (Sigma-Aldrich Co.), GPx 1/2 (B-6) 1 : 500 (Santa Cruz Biotechnology Inc.), GSTP 1 : 500 (Santa Cruz Biotechnology Inc.), GCLC 1 : 500 (Sigma-Aldrich Co.), *β*-actin 1 : 1000 (Santa Cruz Biotechnology Inc.), Lamin B1 1 : 1000 (Santa Cruz Biotechnology Inc.), anti-mouse IgG HRP 1 : 1000 (Sigma-Aldrich Co.), and anti-rabbit IgG HRP 1 : 1000 (Sigma-Aldrich Co.).

### 2.6. Statistical Analysis

Data are expressed as the mean ± standard deviation for each group. The differences among multiple experimental groups were detected by one-way Analysis of Variance (ANOVA) followed by Bonferroni's multiple comparison test. A *P* value of less than 0.05 was considered as significant.

## 3. Results

### 3.1. Plasma Corticosterone Level

**Figure 1:**
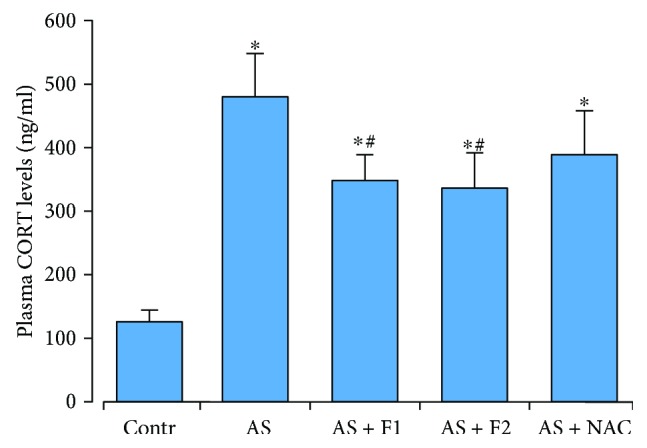
Effect of C_60_FAS (50 *μ*g/kg (F1) and 500 *μ*g/kg (F2)) and NAC on plasma corticosterone levels after acute restraint stress exposure (AS). Values are means ± SD (*n* = 8). The data were analyzed for statistical significance using ANOVA followed by the Bonferroni post hoc test. ^∗^*P* < 0.05 vs. the control group; ^#^*P* < 0.05 vs. the stress group (AS).

The results of plasma corticosterone levels determined in controls and all stressed rat groups are presented in Figure 1. Monitoring changes in the corticosterone level may serve as an indicator of stress response [4, 16]. Acute stress caused a remarkable increase in the plasma corticosterone level (approximately 3–8-fold higher than the level control group, *P* < 0.05), whereas pretreatment with C_60_FAS (50 and 500 *μ*g/kg) significantly decreased the release of plasma corticosterone (by 28 and 32%, respectively) compared to the AS groups alone (*P* < 0.05). NAC administration showed a reduction in the corticosterone level of 19% in comparison with the AS group (*P* > 0.05). There were no significant differences in corticosterone concentration between the controls and any drug treatment alone groups (data not shown).

### 3.2. Oxidative Status of the Brain Tissue after Drug Supplementation and Acute Stress Exposure

**Figure 2:**
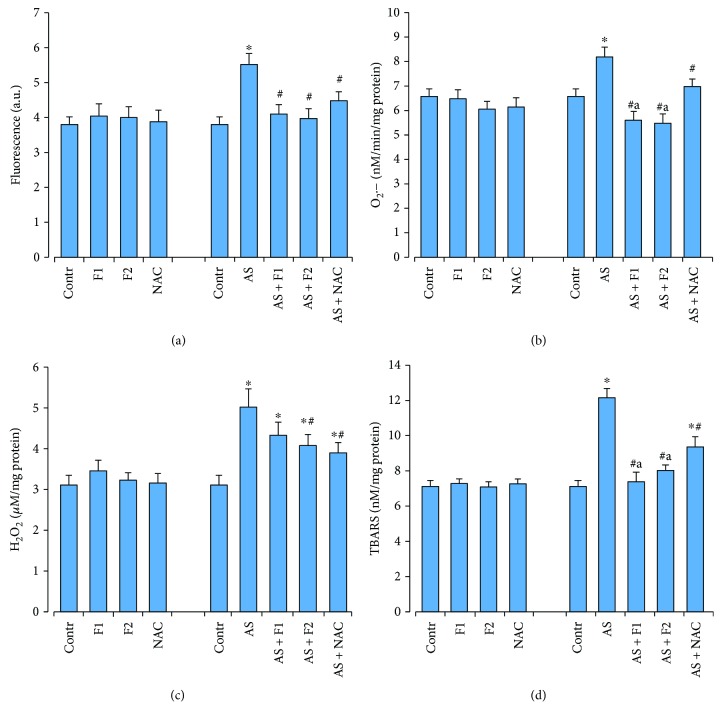
Effect of C_60_FAS (50 *μ*g/kg (F1) and 500 *μ*g/kg (F2)) and NAC on oxidative stress markers: ROS formation (a), O_2_·^−^ (b), H_2_O_2_ (c), and TBARS (d) production in brain tissue after alone administration and after acute restraint stress exposure (AS). Values are means ± SD (*n* = 8). The data were analyzed for statistical significance using ANOVA followed by the Bonferroni post hoc test. ^∗^*P* < 0.05 vs. control group; ^#^*P* < 0.05 vs. acute stress group; ^a^*P* < 0.05 vs. AS + NAC group.

**Figure 3:**
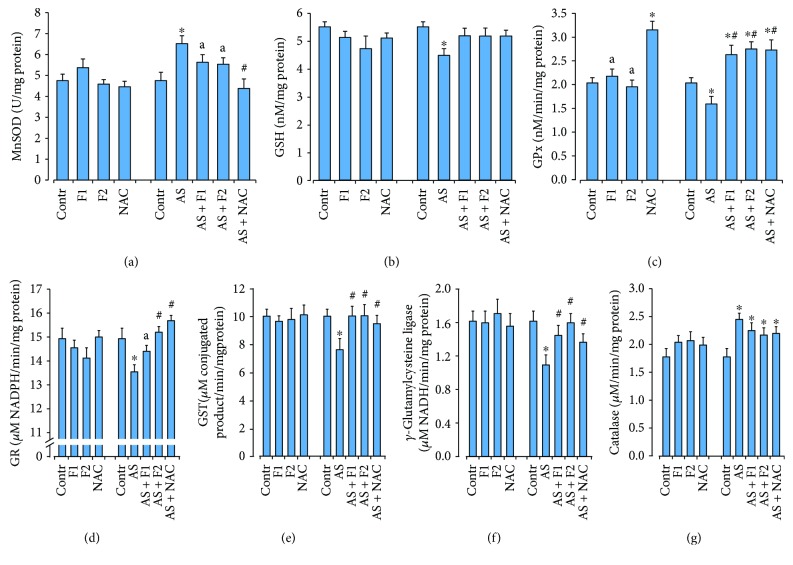
Effect of C_60_FAS (50 *μ*g/kg (F1) and 500 *μ*g/kg (F2)) and NAC on MnSOD activity (a), GSH content (b), and activity of GSH-related enzymes GPx (c), GR (d), GST (e), *γ*-GCL (f), and catalase (g) in the cytosol fraction of brain tissue after alone administration and after acute restraint stress exposure (AS). Values are means ± SD (*n* = 8). The data were analyzed for statistical significance using ANOVA followed by the Bonferroni post hoc test. ^∗^*P* < 0.05 vs. control group; ^#^*P* < 0.05 vs. acute stress group; ^a^*P* < 0.05 vs. AS + NAC group.

Exposure to acute restraint stress significantly elevated the level of ROS generation and O_2_·^−^ and H_2_O_2_ productions by 45, 25, and 60%, respectively (*P* < 0.05) as well as the TBARS accumulation (by 71%), which are the secondary products of lipid peroxidation in comparison with the control rats (*P* < 0.05) (Figure 2). These changes were accompanied by a significant increase in MnSOD and catalase activities by ~37–38% (*P* < 0.05). At the same time, activity levels of GSH-related enzymes (GPx, GR, *γ*-GCL, and GST) were lower by 20–24% (*P* < 0.05) than those in the control rats (*P* < 0.05). Because of the above events, the level of reduced glutathione was diminished (by 19%, *P* < 0.05) (Figure 3). Supplementation of C_60_FAS and NAC before acute stress exposure significantly reduced the free radical level (ROS and O_2_·^−^ generation), H_2_O_2_ concentration, and the intensity of lipid peroxidation as well as increased GSH content compared to the AS alone group as shown in Figures 2 and 3. We have found that C_60_FAS, in both applied doses, prevented the superoxide anion production to a great extent and was more efficient for correction of lipid peroxidation in brain tissue in comparison with NAC (*P* < 0.05). Pretreatment with C_60_FAS (in both concentrations) as well as NAC diminished stress-induced overactivation of MnSOD. All drugs caused significant induction in GSH content and activity of GSH-related enzymes, and these effects were similar. For GR activity, C_60_FAS (500 *μ*g/kg) and NAC administration significantly increased the activity of this enzyme in comparison with C_60_FAS (50 *μ*g/kg). NAC application decreased MnSOD activity to the control level and was more effective than C_60_FAS (in both doses) pretreatment.

### 3.3. Oxidative Status of the Heart Tissue after Drug Supplementation and Acute Stress Exposure

**Figure 4:**
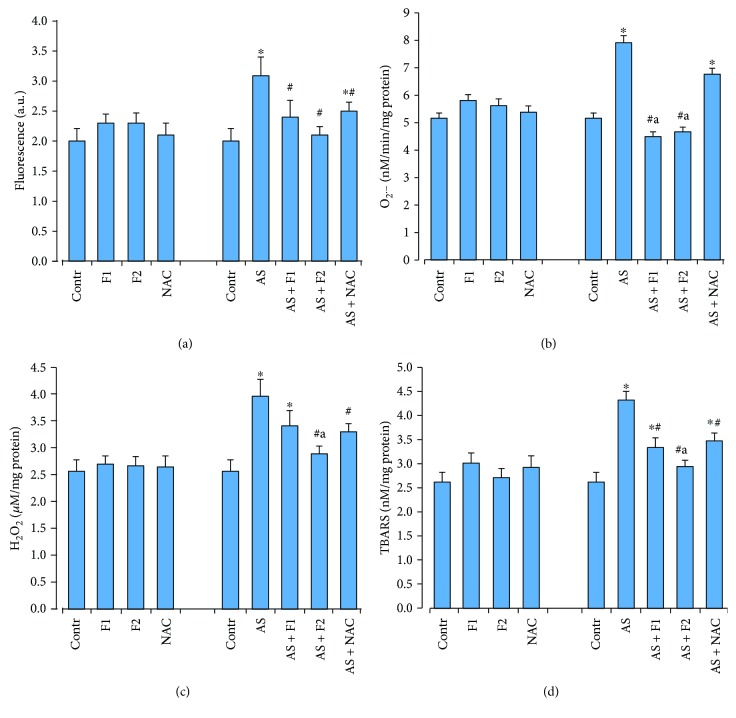
Effect of C_60_FAS (50 *μ*g/kg (F1) and 500 *μ*g/kg (F2)) and NAC on oxidative stress markers: ROS formation (a), O_2_·^−^ (b), H_2_O_2_ (c), and TBARS (d) production in heart tissue after alone administration and after acute restraint stress exposure (AS). Values are means ± SD (*n* = 8). The data were analyzed for statistical significance using ANOVA followed by the Bonferroni post hoc test. ^∗^*P* < 0.05 vs. control group; ^#^*P* < 0.05 vs. acute stress group; ^a^*P* < 0.05 vs. AS + NAC group.

**Figure 5:**
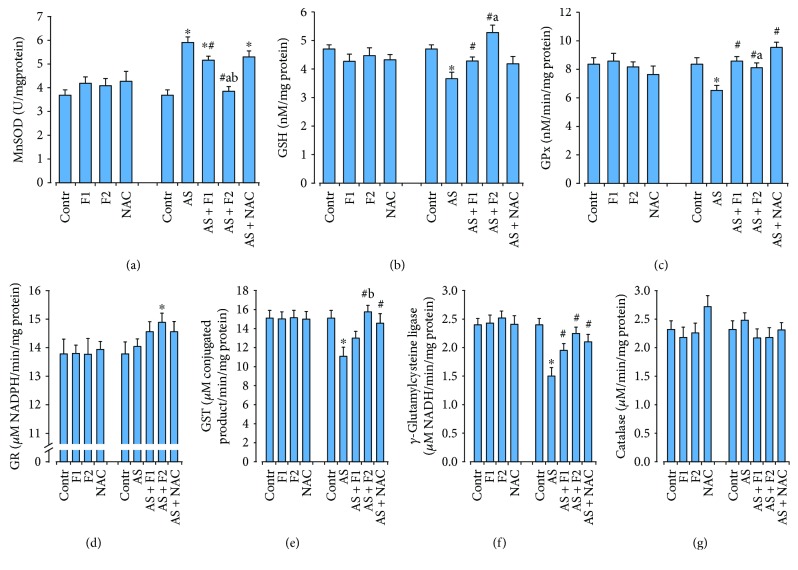
Effect of C_60_FAS (50 *μ*g/kg (F1) and 500 *μ*g/kg (F2)) and NAC on MnSOD activity (a), GSH content (b), and activity of GSH-related enzymes GPx (c), GR (d), GST (e), *γ*-GCL (f), and catalase (g) in the cytosol fraction of heart tissue after alone administration and after acute restraint stress exposure (AS). Values are means ± SD (*n* = 8). The data were analyzed for statistical significance using ANOVA followed by the Bonferroni post hoc test. ^∗^*P* < 0.05 vs. control group; ^#^*P* < 0.05 vs. acute stress group; ^a^*P* < 0.05 vs. AS + NAC group; ^b^*P* < 0.05 vs. AS + F1 group.

Acute restraint stress stimulated ROS and O_2_·^−^ productions (by 55 and 53%, respectively) and increased H_2_O_2_ and TBARS levels (by 54 and 62%, respectively) in relation to the control rats (*P* < 0.05) (Figure 4). In turn, in the heart tissue in response to these changes, there was a significant decrease in the activity and content of endogenous antioxidants, including GPx (by 22%), *γ*-GCL (by 38%), GST (by 27%), and GSH (by 23%) with concomitant increase in MnSOD activities (by 60%) compared to the control groups (*P* < 0.05). No significant changes in GR or catalase activities were observed (Figure 5).

Pretreatment with C_60_FAS (50 and 500 *μ*g/kg) and NAC before stress exposure induced the reduction of the lipid peroxidation because of the inhibition of ROS generation and superoxide anion and H_2_O_2_ releases which we registered in cardiomyocytes. As shown in Figure 4, C_60_FAS (50 and 500 *μ*g/kg) decreased ROS formation (by 22 and 32%), O_2_·^−^ production (by 43 and 35%), H_2_O_2_ concentration (by 15 and 27%), and TBARS content (by 23 and 32%), respectively, compared with the AS alone group (*P* < 0.05). A similar dynamic has been revealed in changes in the above parameters in the NAC treatment group. However, the degree of their inhibition was less than that in the group with C_60_FAS administration, especially after C_60_FAS (500 *μ*g/kg) injection. Applications of C_60_FAS (in both doses) and NAC elevated GSH content (*P* < 0.05) and activities of GPx, GST, and *γ*-GCL (*P* < 0.05) in comparison with the AS rats, reversed the MnSOD overactivation (*P* < 0.05), and maintained GR and catalase activities on the optimal control level (Figure 5).

Interestingly, C_60_FAS (500 *μ*g/kg) was more successful in preventing oxidative stress in heart tissue than administration of C_60_FAS at a lower concentration and NAC. This applied dose of C_60_FAS decreased TBARS, O_2_·^−^, and H_2_O_2_ levels as well as enhanced GSH content and reduced overactivation of MnSOD to a greater extent than did C_60_FAS (50 *μ*g/kg) and NAC (*P* < 0.05).

### 3.4. Effect of C_60_FAS Supplementation and Acute Stress Exposure on Protein Expression of Nrf2, MnSOD, and GSH-Related Enzymes in Brain and Heart Tissues

Nrf2 is a critical transcription factor that tightly controls cellular defense to oxidative stress [34]. Therefore, we first attempted to examine the effects of C_60_FAS supplementation alone and after acute restraint stress on Nrf2 protein content in the nuclear and cytosolic fractions of different rat tissues. Because the nuclear Nrf2 level, specifically, is a direct reflection of ARE-mediated transcriptional activity [32], we examined the cytosolic protein expression of crucial downstream genes such as MnSOD, GPx, GSTP, and GCLC regulated by Nrf2.

**Figure 6:**
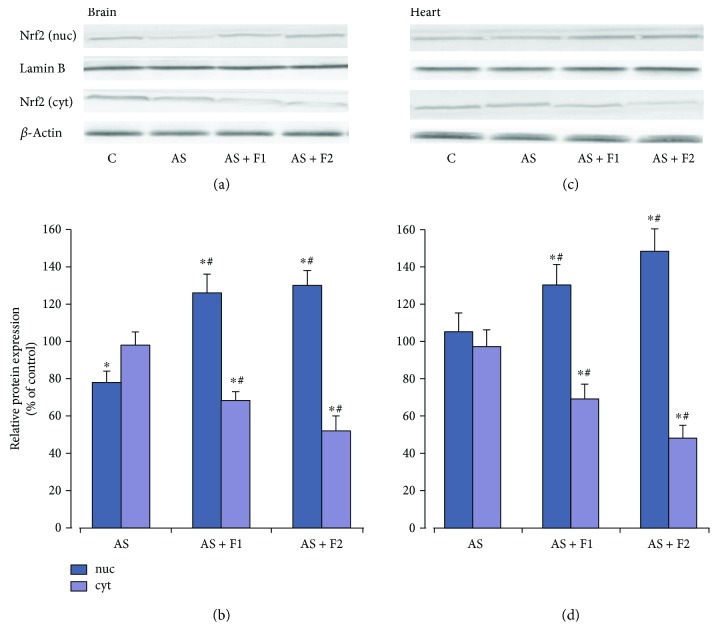
Changes in the nuclear and cytosolic Nrf2 protein expressions in brain and heart tissues after pretreatment of C_60_FAS (50 *μ*g/kg (F1) and 500 *μ*g/kg (F2)) and acute restraint stress exposure (AS). (a and c) Representative western blot and (b and d) densitometric analysis of Nrf2 protein content. Protein extracts were separated by performing SDS PAGE and subsequently electroblotted onto PVDF membranes. The values of the nuclear and cytosolic Nrf2 proteins were normalized to Lamin B and *β*-actin, respectively. Final western blot figured as the histogram is expressed as mean percentages (±SD) over control values from two independent experiments. The control values are taken as 100%. Statistically significant differences are indicated as ^∗^*P* < 0.05 vs. control; ^#^*P* < 0.05 vs. AS.

**Figure 7:**
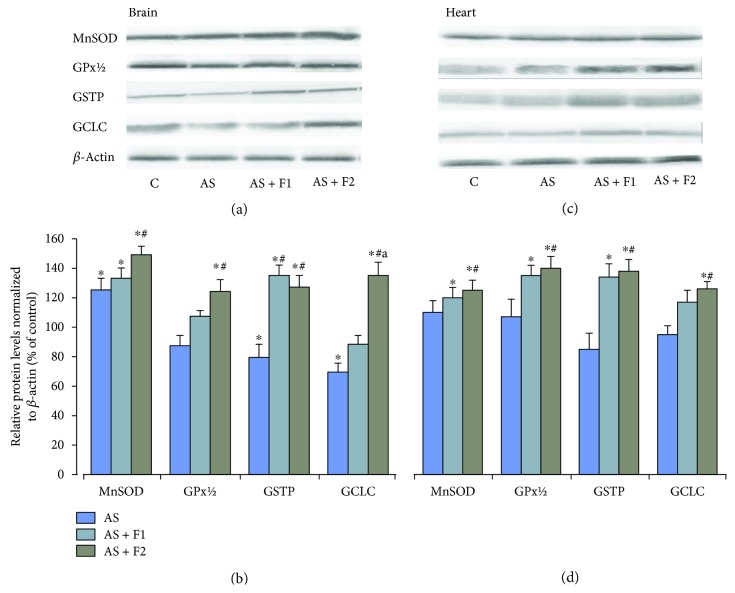
Changes in the MnSOD, GPx 1/2, GSTP, and GCLC protein expressions in brain and heart tissues after pretreatment of C_60_FAS (50 *μ*g/kg (F1) and 500 *μ*g/kg (F2)) and acute restraint stress exposure (AS). (a and c) Representative western blot and (b and d) densitometric analysis of protein levels. Protein extracts were separated by performing SDS PAGE and subsequently electroblotted onto PVDF membranes. Final western blot figured as the histogram is expressed as mean percentages (±SD) over control values from three independent experiments. The control values are taken as 100%. Statistically significant differences are indicated as ^∗^*P* < 0.05 vs. control; ^#^*P* < 0.05 vs. AS; ^a^*P* < 0.05 vs. AS + F1.

Restraint exposure results in decrease (by 22%) in Nrf2 nuclear protein levels in the brain, with a concomitant increase in cytosolic Nrf2 protein levels (*P* < 0.05). In contrast, in the heart, no significant changes in these indices were observed (Figure 6). As shown in Figure 7, in the brain, acute stress triggered a decrease in the protein content of GCLC and GSTP by 30 and 20%, respectively, (*P* < 0.05). The GPx protein content has only the tendency to decrease, but the MnSOD protein level was significantly enhanced (by 26%, *P* < 0.05) in comparison with the control group. These findings correlate with the reduction in GSH content (Figure 3), suggesting that the Nrf2 pathway takes part in GSH synthesis in the brain tissues under AS conditions. At the same time, the heart protein expression of MnSOD and GSH-related enzymes did not show significant changes in comparison with the control group. We found that C_60_FAS (50 *μ*g/kg and 500 *μ*g/kg) administration before AS caused a significant elevation in the Nrf2 protein level in both the brain and heart nuclear extracts in comparison with the control and AS alone groups (*P* < 0.05), which was accompanied by decreases in the Nrf2 cytosolic protein expression (*P* < 0.05) indicating translocation of Nrf2 from cytosol to the nucleus. Together with the increase in nuclear Nrf2 levels, we registered a statistically significant increase in the protein expression of MnSOD, GPx, GSTP, and GCLC in the brain as well as in the heart cytosolic fractions relative to the AS alone and control groups (*P* < 0.05). The above indices are expressed to a greater degree after supplementation of C_60_FAS at a higher dose.

### 3.5. Effect of Drug Administration on Prooxidant/Antioxidant Balance and Protein Expression in Rat Tissues in a Normal Physiological State

**Figure 8:**
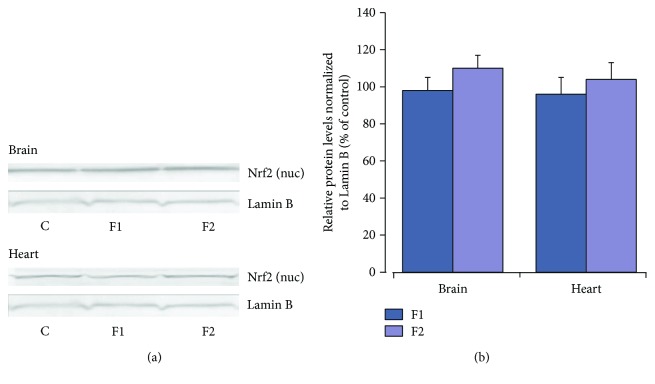
Changes in the nuclear Nrf2 protein expression in brain and heart tissues after pretreatment of C_60_FAS (50 *μ*g/kg (F1) and 500 *μ*g/kg (F2)). (a) Representative western blot and (b) densitometric analysis of Nrf2 protein content. Protein extracts were separated by performing SDS PAGE and subsequently electroblotted onto PVDF membranes. Final western blot figured as the histogram is expressed as mean percentages (±SD) over control values from two independent experiments. The control values are taken as 100%.

**Figure 9:**
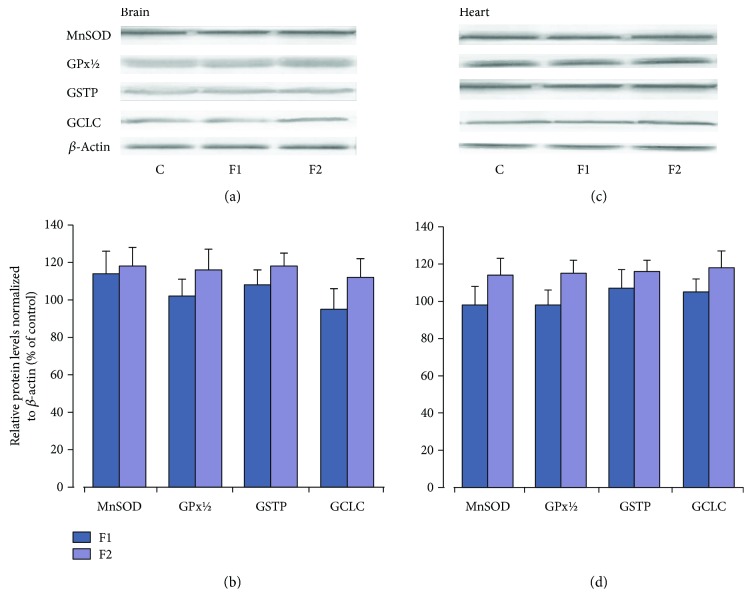
Changes in the MnSOD, GPx 1/2, GSTP, and GCLC protein expressions in brain and heart tissues after pretreatment of C_60_FAS (50 *μ*g/kg (F1) and 500 *μ*g/kg (F2)). (a and c) Representative western blot and (b and d) densitometric analysis of protein levels. Protein extracts were separated by performing SDS PAGE and subsequently electroblotted onto PVDF membranes. Final western blot figured as the histogram is expressed as mean percentages (±SD) over control values from two independent experiments. The control values are taken as 100%.

When the investigated drugs were applied alone in the brain and heart tissues, there were no changes in oxidative stress markers or indices of antioxidant defense systems except GPx activity, which in the brain tissue after NAC administration was significantly raised in comparison with the control and C_60_FAS-treated rats (*P* < 0.05) (Figures 2–5). The effect of C_60_FAS administration alone on the Nrf2 protein expression is depicted in Figure 8. In both tissues, the protein level of Nrf2 in the nuclear level was close to the control level (*P* > 0.05) as well as in cytosol (data not shown). Protein content of MnSOD and GSH-related enzymes in both tissues tended to increase, but this effect had no statistical significance (Figure 9).

## 4. Discussion

Although several previous studies have shown that C_60_ can protect cells against oxidative stress-induced cell death by activating the Nrf2/ARE pathway in vitro on cell cultures [28, 29], it remains unclear whether C_60_ could have the same effect on oxidative stress in vivo in animal models. In this study, as the first step towards understanding the protective role of Nrf2 activation, we studied the effect of C_60_FAS in two doses and NAC as the positive control, on prooxidant/antioxidant homeostasis in rats that were subjected to acute restraint stress.

In the present study, 6 h of restraint stress markedly increases ROS, O_2_·^−^, and H_2_O_2_ productions and the consequent LPO intensification that induces disorders in antioxidant status of the brain and the heart tissues, in accordance with many studies showing that restraint stress significantly elevates oxidative status and increases or decreases antioxidant enzyme activities in different rat tissues, depending on the severity and duration of the restraint stress protocol [12, 13, 15]. Indeed, previous studies have already shown that immobilization stress targets the brain for lipid peroxidation, as the levels were found to be highest in this tissue [14, 49].

We found that AS significantly altered activity of SOD and GPx, two of the key enzymes that are a first line of antioxidant defense and function in concert to prevent ROS reactions in response to oxidative stress. Overactivation of MnSOD without a concomitant increase in GPx activity, which we demonstrated in our study, results in the accumulation of H_2_O_2_ that not only changes the cellular redox status but also participates in the Fenton reaction, leading to production of noxious hydroxyl radicals [5]. A significant diminution in GSH content and GSH-related enzymes denotes disorders in GSH biosynthesis because of the absence of an adequate antioxidative defense in rat tissues after restraint exposure [8, 9]. In our study, together with intensification of the oxidative process, we registered a significantly elevated level of plasma corticosterone, which is an important indicator of the stress condition. Stress is known to activate the hypothalamic–pituitary–adrenal axis, which results in an increased secretion of glucocorticoids that affect not only the brain but also the peripheral organs [16]. A high level of glucocorticoids may affect the redox status of tissues via different mechanisms, including an increase in superoxide cell production and the impairment of tissue antioxidant capacity [49]. Previous reports show that high levels of corticosterone decrease the activity of the key antioxidant enzyme glutathione peroxidase, directly reducing the total glutathione pool as well as the NADPH levels, required for regeneration of GSH from oxidized glutathione [17, 50]. The increase in the levels of circulating corticosterone after acute immobilization was demonstrated to be directly proportional to the increase in oxidative mediators as well as injuring neuro- and cardiosystems [51]. All these findings permit us to suggest that the increased corticosterone level during our stress model may in addition affect the antioxidant capacity in both rat tissues that were investigated.

Under these circumstances, the use of different antioxidants and agents with free radical scavenging properties to counteract the noxious events elicited by acute stress represents an effective therapeutic strategy mostly employed to handle oxidative damage [52]. Among these strategies, carbon nanoparticles with intrinsic ROS scavenging and antioxidant properties exhibit superior pharmacological features due to enhanced absorption and bioavailability [53].

We have shown that 1 week of treatment with C_60_FAS (50 and 500 *μ*g/kg) before restraint stress exposure induces the reduction of the lipid peroxidation intensity because of the inhibition of ROS production, namely, superoxide anion release and H_2_O_2_ generation, which we registered in cardiomyocytes and brain cells. The mitochondrial respiratory chain and enzymatic reactions by NADPH oxidase, xanthine oxidase, cyclooxygenases, and lipoxygenase are known to be an important endogenous source of ROS under stress conditions [7]. Superoxide anion is a common precursor of ROS that is quickly involved in two metabolic pathways: rapid conversion into hydrogen peroxide and oxygen by superoxide dismutase and generation of highly toxic peroxynitrite via reaction with nitric oxide [5]. We observed that C_60_FAS application (in both doses investigated) drops the hyperactivity of MnSOD and therefore the production of superoxide anion, which serves as the substrate for this enzyme and designate of MnSOD activity. The consequence of this activity was a decrease in the level of peroxide in the brain cells and cardiomyocytes. Although MnSOD is a mitochondrial enzyme, we detected the MnSOD protein in the cytosolic fraction. Since MnSOD is encoded in the nucleus, synthesized as a precursor in the cytoplasm, and transported to the mitochondria through a mitochondrial targeting sequence, the measured cytosolic MnSOD level may illustrate its transit to the mitochondria after nuclear synthesis [54]. Thus, an increased cytosolic MnSOD protein level could be derived from mitochondrial MnSOD and/or inappropriate transport of newly synthesized MnSOD into mitochondria. In any case, the appearance of MnSOD in the cytosolic fraction clearly indicates a loss of brain and heart mitochondrial membrane integrity under the stress condition. Our findings that C_60_FAS affects the MnSOD activity suggest that C_60_FAS has an unlimited ability to penetrate biological barriers with subsequent easy access to subcellular compartments, as was reported earlier [55].

Fullerene and its derivatives are a powerful antioxidant due to the delocalization of the *ð*-electrons over the carbon cage, which can readily react with ROS, in particular superoxide anion, hydroxyl, and lipid radicals. Numerous studies have demonstrated the free radical scavenging capabilities to such a degree that fullerenes have been described as “free radical sponges” [19, 26, 28]. Fullerene is postulated to act as a peculiar antioxidant that does not only react directly with free radicals but also initiate and catalyse the reaction of ROS recombination (dismutation) occurring in ordered interfacial water shells near the fullerene's surface. This assumption could explain why fullerene exhibits prolonged bioactive effects, including antioxidant capacity, even in small and supersmall concentrations [20].

GSH homeostasis is known to be regulated by the following three aspects: (a) GSH is synthesized from glutamate, glycine, and cysteine by glutathione synthetase and *γ*-glutamylcysteine ligase; (b) GSH is oxidized to GSSG by the activity of GPx, thus regulating and maintaining the cell redox status; (c) GSSG is reduced to GSH with the mediation of GR, by which is a substantial part of the intracellular GSH recycling [9]. GSH-related enzymes such as GPx, GR, GST, and *γ*-GCL are pivotal enzymes in the regulation of intracellular redox status through modulation of glutathione homeostasis and upregulation through the Nrf2-Keap1-ARE pathway [8, 52].

We found that 6 h restraint caused a decrease in the GSH level and GCL activity in brain cells that indicates disorders in the GSH-biosynthetic cycle. The decreased activity in glutathione reductase induces the accumulation of oxidized glutathione in the brain, which is indicative of the severity of the oxidative stress [8]. In addition to peroxide-mediated inactivation of GPx, superoxide anion and peroxynitrite overproductions can inhibit GPx activity [56] as was obtained in our study. A previous study by Atif et al. [15] demonstrated that the activity of both GR and GPx enzymes is decreased in the hippocampus following similar stress durations. In the heart, we demonstrated decrease in *γ*-GCL and GPx activities without changes in GR activity. In both tissues, we registered decrease in GST activity, which correlated with GST protein content. At the same time, the activity of CAT, the main scavenger of H_2_O_2_ in the neurons, was increased in the brain and had a tendency to increase in the heart that agreed with early reports [57]. GSTs are ubiquitous, multifunctional enzymes that detoxify endogenous and exogenous electrophiles, including epoxides, aldehydes, and peroxides [58]. Therefore, the decrease in GST that was observed after acute stress in rat tissues may induce additional intoxication by harmful LPO byproducts.

Various tissues are known to have different sensitivities to oxidative stress challenges depending on several factors such as oxygen consumption, metabolic rates, susceptibility to oxidants, and antioxidant levels. In the present study, after analysis of the oxidative stress markers, we established that the brain, in contrast to the heart, was more vulnerable to oxidative stress in our experimental model.

C_60_FAS supplementation showed the increase in GSH content as well as GPx, GST, and GR activities in both tissues investigated in comparison with the AS alone group promoted coordinated action GSH redox-cycle enzymes and involvement of GSH in the metabolism of toxic aldehydes and peroxides with a decrease in cell injuries. The increased GCL activity suggests the involvement of C_60_ in modulating glutathione biosynthesis under AS condition. C_60_FAS administration induced some decrease in CAT activity in both tissues compared with the AS alone group with normalization of GPx activity indicating the decrease in the intensity of peroxidation processes. Similar reports on several other stresses such as doxorubicin intoxication and irradiation showed that fullerene administration reduces ROS generation and lipid peroxidation and increases the GSH level and activity of GSH-related enzymes [23, 24]. In our study, we used NAC as a positive control during the investigation of cell prooxidant/antioxidant homeostasis because this drug is an antioxidant that potentially reduces the damaging effect of oxidative stress [36]. NAC exerts its effect both as a source of sulphhydryl groups (preventing GSH depletion) and as a free radical scavenging agent through a direct reaction with highly oxidizing radicals such as ·OH, ·NO_2_, and CO_3_·^−^ [33]. Treatment with NAC inhibited lipid peroxidation, increased catalytic activity of glutathione peroxidase, glutathione reductase, and superoxide dismutase, and replenished GSH levels in animal tissues under different pathological conditions [59, 60].

We observed that C_60_FAS pretreatment provided sufficiently strong antioxidant defense with a kind of effect like the effect of NAC. The C_60_FAS (500 *μ*g/kg) was more effective in preventing oxidative stress disorders than NAC and C_60_FAS at the concentration of 50 *μ*g/kg.

Neuro- and cardioprotective actions of C_60_FAS under restraint stress were also confirmed by a significant decrease in corticosterone concentration, as demonstrated in our study. The reduction markers of oxidative stress and normalization antioxidant activity suggest that C_60_FAS may induce heart and brain tissues to maintain sufficient antioxidant capacity against ongoing oxidative stress.

Free radical metabolites generated during extreme influence are known to function as signaling molecules activating transcription factors and specific genes that result in de novo protein synthesis, including antioxidant enzymes. These molecular mechanisms might be substantial for triggering of adaptive reactions and forming protective mechanisms against oxidative stress [32].

To further investigate the potential mechanisms of C_60_FAS-mediated protection against oxidative stress, we examined the protein expression of Nrf2, which has demonstrated a pivotal role in cell protection by the coordinate transcription of ARE-regulated antioxidant genes, including GPx2, GST, *γ*-GCLC, GR, and SOD [52].

In the present study, exposure to AS induced some decrease in the Nrf2 nuclear protein content in the brain with concomitant increase in the cytosol fraction. Our results agreed with previous findings showing significant decrease in Nrf2 nuclear protein in the whole brain tissue after 6 h of restraint stress [12, 18], in the rat hippocampus, without changes in the prefrontal cortex after acute immobilization for 30 min [61]. Spiers et al. [14] reported a transiently decreased nuclear Nrf2 that coincided with increased expression of 11*β*-HSD1-corticosterone regenerating enzyme in the hippocampus of rats subjected to 4 h restraint stress.

Numerous factors are involved in Nrf2 regulation at both transcriptional and posttranscriptional levels and affect its distribution in cell compartments [30]. On the one hand, Nrf2 belongs to the stress-responsive transcription factors that are largely controlled at the level of protein stability. Nrf2 has been found to be an unstable protein, due to the proteolytic degradation through the ubiquitin-dependent pathway. Under basal conditions, Keap1 continuously drives the regulation of Nrf2 maintaining low cellular levels of the protein. However, under oxidative stress, the regulation of Nrf2 becomes complex, involving both Keap1-dependent and -independent mechanisms [31]. On the other hand, there are alternative mechanisms of Nrf2 regulation, including phosphorylation of Nrf2 by various protein kinases (PKC, PI3K/Akt, GSK-3b, and JNK), interaction with other protein partners (p21 and caveolin-1), and epigenetic factors (micro-RNAs-144, -28, and -200a and promoter methylation) [30]. Thus, Keum et al. demonstrated that p38 phosphorylates Nrf2 and promotes its association with Keap1, thereby preventing its nuclear translocation. Besides, prolonged inflammation induces activation of specific kinases and epigenetic modification, which blocks the Nrf2 and contribute to neurological injury [62]. In addition, Ki et al. [63] showed that the activated glucocorticoid receptor modulates Nrf2 signaling and alters Nrf2 target gene expression in the brain through binding of the glucocorticoid receptor to its glucocorticoid response element. Under stress conditions, glucocorticoids can suppress cellular antioxidant defense capacity by impairing Nrf2-dependent antioxidant response [17]. Furthermore, NF-*κ*B is a negative regulator of NRF2 by binding to response elements in the NRF2 gene promoter [64]. As was shown, the p65 subunit can block NRF2 binding to CREB protein and recruit histone deacetylase 3, a corepressor of ARE [65]. These pathways can be regulated by acute stress and could be implicated, at least in part, in the effects reported here.

In parallel with loss in Nrf2 protein content in the brain nuclear fraction, we observed some decease in the protein level of GCLC (*P* < 0.05), GPx (*P* > 0.05), and GSTP (*P* < 0.05) after acute stress exposure. Although MnSOD is a target of Nrf2 activation, poor Nrf2 function did not correspond to MnSOD protein expression in our study where we found significant enhancement in MnSOD protein content (*P* < 0.05). Such a difference could be due to alternative signaling pathways operating in the brain that upregulated MnSOD. MnSOD is known to be an NF-*κ*B-related protein, and under stress conditions, NF-*κ*B is actively involved in the expression of MnSOD [66]. In addition, a high level of H_2_O_2_ generation as product of MnSOD catalytic activity, which we observed in our study after stress exposure, may act as a signaling molecule in the NF-*κ*B activation [67].

No statistically significant changes were observed in nuclear and cytosol Nrf2 protein accumulations as well as in protein expression of Nrf2-target genes MnSOD, GPx, and GCL except GST in the myocardium of rats that were subjected to restraint stress.

The present study showed that C_60_FAS administration induced significant enhancement of nuclear Nrf2 protein in the brain and the myocardium of rats subjected to restraint stress for 6 h with concomitant decrease in the cytosol fraction. The levels of protein expression of GSH-related enzymes positively correlated with Nrf2, suggesting that the upregulation of GCLC, GST, and GPx may depend on the Nrf2/ARE pathway. We assume that C_60_FAS effects on GSH recycle via induction of *γ*-GCL as well as GPx and GST protein expressions, and this is necessary and sufficient to reestablish GSH system homeostasis.

In the brain, the MnSOD protein level continued to increase in comparison with AS. In contrast, in the heart, this index remains at the level of the control and AS alone groups. These findings confirm that the effect of C_60_FAS application for MnSOD was a tissue specific. Significant modulation of the antioxidative enzymatic system at the protein level that was observed in our study showed a complex network of transcriptional and translational events modified by C_60_FAS during the cellular response to restraint stress.

Recently, Ye et al. [29] have shown that in the *in vitro* model, polyhydroxylated derivatives of fullerene after administration alone enhanced Nrf2 nuclear protein translocation and upregulated mRNA and protein expression of phase II antioxidant enzymes, including HO-1, NADPH: quinine oxidoreductase 1, and *γ*-glutamate cysteine ligase, in 549 cells. By analogy, pretreatment with C_60_(OH)_24_ showed significant protective effects in the 1-methyl-4-phenylpyridinium- (MPP+-) induced acute cellular Parkinson disease model in human neuroblastoma cells through increase in expression of Nrf2 and expression and activity of *γ*-glutamyl cysteine ligase and the level of glutathione [28]. In contrast, in our study, in the *in vivo* model when C_60_FAS (50 and 500 *μ*g/kg) was applied in a normal physiological state, the western blot analysis showed only a tendency to increase without statistically significant changes in protein expression of MnSOD and GSH-related enzymes of both tissues that correlated with Nrf2 protein content in the nuclear and cytosol fractions. Similarly, C_60_FAS (50 and 500 *μ*g/kg) applied alone did not induce any changes in investigated oxidative stress markers and antioxidant enzyme activity in the brain and the heart tissues. All these facts allow us to assume that *in vivo*, C_60_FAS did not affect the endogenous antioxidant status of the rat tissues in the absence of any stress conditions.

## 5. Conclusions

In conclusion, our results suggest that the neuro- and cardioprotection of C_60_FAS are mediated, on the one hand, by direct removal of ROS, namely, superoxide anion and hydrogen peroxide, and thus decrease lipid peroxidation, on the other hand, by the effect on rat tissue antioxidant capacity. Under stress condition, C_60_FAS administration promotes Nrf2 nuclear accumulation and triggers the protein expression of a panel of antioxidant and phase II enzymes, at least partially, through the Nrf2/ARE signaling pathway. We suggest that C_60_FAS affects endogenous glutathione homeostasis by modulation of glutathione biosynthesis as well as antiperoxide defense via upregulation of activity and protein expression of GSH-related enzymes *γ*-GCL, GPx, and GST. Under stress exposure, C_60_FAS strengthens antiradical defense through upregulation of MnSOD in brain cells and maintains MnSOD protein content at the control level in the myocardium. C_60_FAS supplementation has dose-dependent and tissue-specific effects. These results suggest that the use of C_60_FAS may be of interest as a potential therapeutic strategy in correction of oxidative stress-based stress conditions.

## Data Availability

The biochemical data used to support the findings of this study are available from the corresponding author upon request.

## Abbreviations

C_60_FAS: Pristine C_60_ fullerene aqueous colloid solution
ROS: Reactive oxygen species
O_2_·^−^: Superoxide anion
H_2_O_2_: Hydrogen peroxide
GST: Glutathione-S-transferase
GPx: Glutathione peroxidase
*γ*-GCL: *γ*-Glutamate-cysteine ligase
GR: Glutathione reductase
GSH: Reduced glutathione
MnSOD: Manganese superoxide dismutase
Nrf2/ARE: NF-E2-related factor 2/antioxidant response element.
